# Spatiotemporal mapping of RNA editing in the developing mouse brain using in situ sequencing reveals regional and cell-type-specific regulation

**DOI:** 10.1186/s12915-019-0736-3

**Published:** 2020-01-14

**Authors:** Elin Lundin, Chenglin Wu, Albin Widmark, Mikaela Behm, Jens Hjerling-Leffler, Chammiran Daniel, Marie Öhman, Mats Nilsson

**Affiliations:** 1 Department of Biochemistry and Biophysics, Science for Life Laboratory, Stockholm University, SE-171 21 Solna, Sweden; 2 Department of Molecular Biosciences, The Wenner-Gren Institute, Stockholm University, Svante Arrheniusväg 20C, 106 91 Stockholm, Sweden; 3 German Cancer Center (DKFZ), Im Neuenheimer Feld 280, 69120 Heidelberg, Germany; 4 Laboratory of Molecular Neurobiology, Department of Medical Biochemistry and Biophysics, Karolinska Institutet, Stockholm, Sweden

**Keywords:** Single-cell resolution, RNA editing, Spatially resolved transcriptomics, Brain development

## Abstract

**Background:**

Adenosine-to-inosine (A-to-I) RNA editing is a process that contributes to the diversification of proteins that has been shown to be essential for neurotransmission and other neuronal functions. However, the spatiotemporal and diversification properties of RNA editing in the brain are largely unknown. Here, we applied in situ sequencing to distinguish between edited and unedited transcripts in distinct regions of the mouse brain at four developmental stages, and investigate the diversity of the RNA landscape.

**Results:**

We analyzed RNA editing at codon-altering sites using in situ sequencing at single-cell resolution, in combination with the detection of individual ADAR enzymes and specific cell type marker transcripts. This approach revealed cell-type-specific regulation of RNA editing of a set of transcripts, and developmental and regional variation in editing levels for many of the targeted sites. We found increasing editing diversity throughout development, which arises through regional- and cell type-specific regulation of ADAR enzymes and target transcripts.

**Conclusions:**

Our single-cell in situ sequencing method has proved useful to study the complex landscape of RNA editing and our results indicate that this complexity arises due to distinct mechanisms of regulating individual RNA editing sites, acting both regionally and in specific cell types.

**Electronic supplementary material:**

The online version of this article (10.1186/s12915-019-0736-3) contains supplementary material, which is available to authorized users.

## Background

Adenosine-to-inosine (A-to-I) RNA editing of double-stranded RNA (dsRNA), catalyzed by adenosine deaminase acting on RNA (ADAR) protein family, is an essential event for the proper functioning of the mammalian brain [1]. Inosine forms base-pairs with cytosine and is generally read as guanosine. Thus, editing events have the capacity to diversify neuronal gene expression, including amino acid sequence changes [2, 3] and regulation of alternative splicing [4, 5]. Interestingly, many codon-altering RNA editing sites, recoding editing, are found in transcripts crucial for functional neurotransmission and brain function [6]. The level of recoding editing at a site within a transcript is generally not 100%, so therefore unedited and edited transcripts are present in the same tissue, increasing the proteome diversity which is likely to contribute to the functional complexity of neural cells.

The mammalian ADAR protein family consists of three members: ADAR1, ADAR2, and ADAR3 [7]. ADAR1 and ADAR2 have distinct yet overlapping specificities for editing substrates, resulting from how they are able to interact with specific adenosines while being constrained by the structure of the editing substrates [8]. ADAR3 lacks enzymatic activity but is still a dsRNA-binding protein and therefore it has been suggested to act as an inhibitor of RNA editing [9, 10]. ADAR1 has a vital function in suppressing aberrant activation of the innate immune system [11, 12], while the major function of ADAR2 seems to involve editing of transcripts expressed in the central nervous system [13, 14]. Interestingly, rodent ADAR2 edits its own pre-mRNA, creating an alternative splice site. The alternative splicing, as a result of editing, leads to a frameshift and decreased ADAR2 protein levels. This results in an auto-regulatory loop of ADAR2, where high ADAR2 activity will decrease the expression of active ADAR2 protein until a balance is reached [4, 15]. In addition to this auto-regulatory loop, trans-acting regulators of RNA editing have been identified, whose expression likely contributes to temporal, cell type-specific and tissue-specific RNA editing patterns [16]. These include regulators of ADAR expression and stability [14, 17], subcellular localization [18], activity [19], and regulators of editing at specific sites [20]. Given these many mechanisms for regulating editing levels, generally or for specific substrates, there are many ways to generate a diverse editing landscape.

Next-generation RNA sequencing (RNA-seq) studies have revealed that editing levels at individual sites varies to a large degree in different tissues, and this variation is not simply explained by correlation to the expression of the editing enzymes [14, 21]. These studies are limited in resolution by the ability to isolate specific regions of different tissues. Furthermore, bulk tissue RNA sequencing fails to reveal variation between individual cell types in a tissue. To bypass these limitations, single-cell RNA-seq has recently been used to reveal previously unknown variations in RNA editing between different cell types [22–25]. However, these studies are still limited by the difficulties of cell isolation, lack of sequencing depth, and loss of the spatial organization of the tissue. RNA editing is developmentally regulated in the brain, with the editing levels of most recoding substrates increasing during brain development [21, 26]. However, the limitations of current methods have provided little understanding of the spatiotemporal dynamics of RNA editing during brain development. For instance, it remains unknown in which cell types the developmental increase in editing occurs. More generally, to which degree there are cell-type-specific as well as regional editing patterns and how these are established during brain development remain to be investigated in order to achieve a complete understanding of the role of RNA editing in the developing brain.

To address these challenges, we have analyzed transcripts that are edited and their editing levels in mouse brain sections in situ during development from embryo to adult, providing spatial resolution of A-to-I editing. We applied targeted in situ sequencing (ISS) [27] which employs padlock probes and target-primed rolling circle amplification (RCA) detection providing robust discrimination of single nucleotide variants of transcripts in situ [28]. A panel of barcoded padlock probes was designed to examine recoding A-to-I editing levels in a set of transcripts by in situ sequencing chemistry in a multiplexed fashion [27]. Our results show how the editing of 14 sites changes in a spatial-temporal manner during mouse brain development revealing a previously unknown level of complexity in the regulation of A-to-I RNA editing.

## Results

### Study design and rational

**Fig. 1.**
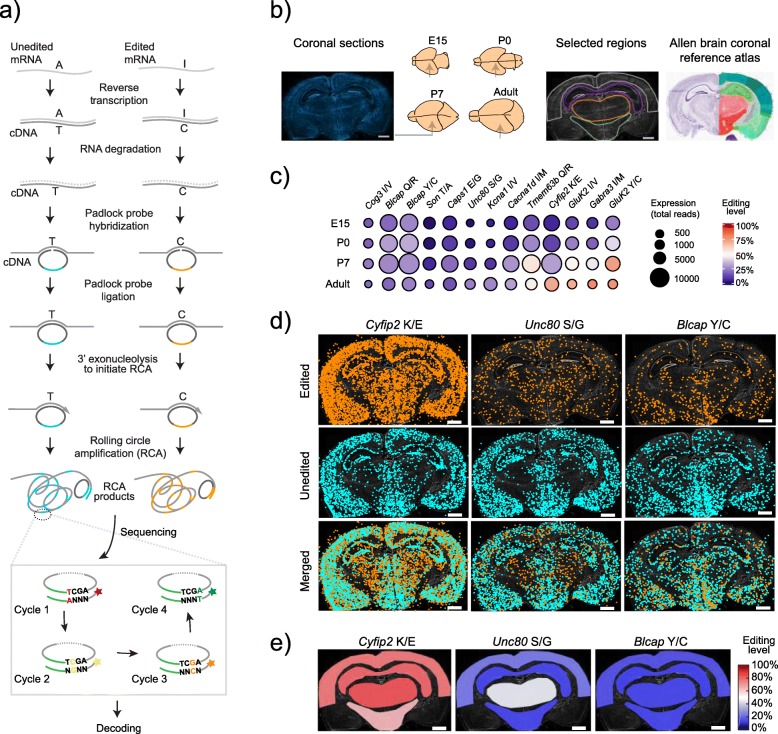
Detection of RNA editing on mouse brain tissue by padlock probes and in situ sequencing. **a** Workflow for the detection of edited and unedited transcripts and sequencing of the barcode by in situ sequencing. **b** Representative image of the DAPI staining of cell nuclei in a coronal section from an adult brain. The arrows in the schematic illustrations of mouse brains at different developmental stages indicate the bregma coordinate from where the coronal sections were obtained. The regions selected for the regional analysis are outlined in adult brain and were selected based on the reference image from the Allen Brain Atlas. Regions of interest (ROIs) were manually outlined in the tissue image for each replicate. **c** Whole brain editing data, the mean of all replicates per developmental stage (Additional file 5), for each edited site in a bubble chart, derived from raw ISS reads in Additional files 7, 8, 9, and 10. The color of the bubbles indicates the editing level and the size of the bubble the level of expression for each transcript. Lowly expressed targets (< 500 reads) were excluded from further analysis and are not shown here. **d** Representative images for the results from one brain section from an adult mouse showing the spatial distributions of edited (orange) and unedited (cyan) transcripts of *Cyfip2*, *Unc80*, and *Blcap*. Each dot represents one transcript. **e** Representative images of the regional editing levels in one brain section from adult mouse for *Cyfip2* K/E, *Unc80* S/G, and *Blcap* Y/C, where the color of the region indicates the level of editing. The scale bar is 1 mm in all images

We studied the pattern of A-to-I RNA editing during development, by analyzing coronal sections from brain tissues from E15, P0, P7, and adult mice (*n* = 5) using in situ sequencing (ISS). The sections were taken at approximately the same bregma coordinate (interaural ≈ 2 mm, bregma ≈ 2 mm for adult brain tissue). Padlock probes (PLPs) were designed to target 22 editing sites including the *Adar2* self-editing site (referred to as *Adar2* auto-editing) (Additional files 1 and 2). We chose recoding editing sites, where editing confers a non-synonymous amino acid substitution. Each edited position was targeted by a pair of PLPs, with one PLP targeting the cDNA of the unedited transcript and the other targeting the cDNA of the edited transcript (Fig. 1a). The PLPs were designed to hybridize with the ligation site at the 5′ side of the edited position; thus, each pair of PLPs differed only in the final base (either A or G) at the 3′ end as well as in the barcode located in the linker sequence (for details, see “Methods”). The PLPs targeting auto-editing of *Adar2* were designed to ligate at the exon junction where editing regulates the splicing. PLPs for the transcripts of the editing enzymes *Adar1*, *Adar2*, and *Adar3* (five probes each, targeting different parts of the transcripts) were also included in the target panel (Additional file 2) to monitor the expression of these ADAR enzymes as well as to connect the expression of these enzymes to individual edited transcripts. To visualize the main cell types in mouse brain, PLPs targeting the transcripts of markers for interneurons (*Sst*, *Pvalb*, *Vip*, and *Cck*), pyramidal neurons (*Pcp4* and *Ndnf*), oligodendrocytes (*Plp1* and *Enpp2*), and astrocytes (*Gfap*) were included (Additional files 2 and 3). After hybridization and ligation, the PLPs were amplified generating micron-sized amplicons that can be analyzed by sequencing by ligation (SBL) chemistry in situ [27] (Fig. 1a). Based on the 2D coronal reference atlas from the Allen Brain Atlas, the regions neocortex, hippocampus, thalamus, and hypothalamus were selected for a regional analysis (Fig. 1b). This was done by manually selecting regions of interest (ROIs) in the tissue image for each replicate and extract data for each ROI for further analysis (Fig. 1b). For E15, “The Atlas of Mouse Development” [29] was used to identify thalamus and hypothalamus. Upon initial analysis of the generated data, we decided to focus on the editing sites for which we obtained a critical number of reads, at least 500, from whole brain for the least prevalent of the edited or the unedited transcript variant. Hence, seven editing sites were excluded from further analysis due to the coverage (Fig. 1c and Additional files 1 and 4). The number of reads for the edited and unedited transcript variants was used to derive the editing level for each individual edited site (Fig. 1c and Additional file 5). The average detection rate ranged from 1.91 to 6.17 reads per cell for most tissue sections, and generally, the read count per cell was higher in P7 and adult tissue sections (Additional files 6, 7, 8, 9, and 10). The read counts for edited and unedited forms of the targeted transcripts were consistent between our biological replicates (Additional file 11), which constituted of sections from corresponding areas and age of different brains and displayed unique spatial distribution patterns for some of the included edited transcripts (Fig. 1d) as well as region-specific editing levels (Fig. 1e).

### Whole brain editing analysis reveals an overall increase in editing in the developing mouse brain

In order to validate the method as a way to quantify RNA editing, the in situ data was evaluated by comparing it with published data generated from RNA-seq analysis. Our analysis of in situ data from brain tissue sections revealed that the editing level increased during development for most of the sites; *Adar2* auto-editing, *Cyfip2* K/E, *Gabra3* I/M, *GluK2* Y/C, *GluK2* I/V, *Kcna1* I/V, *Tmem63b* Q/R, and *Unc80* S/G (the unedited amino acid is written first and the amino acid resulting from editing, second). Other edited positions displayed constant low editing (*Caps1* E/G, *Cog3* I/V, *Blcap* Y/C, and Q/R) (Fig. 1c and Additional files 4 and 5), correlating well with previous results [21, 26]. In the validation of our in situ assay, the editing level of the Q/R site in *Gria2* did not correlate well with previous studies. We believe this discrepancy is due to interference from a known nearby editing site (located four nucleotides downstream of the editing site), which overlaps with the target binding site of the *Gria2* Q/R probes. Thus, this site was excluded from further analysis. Overall, the results from our ISS assay corresponded well to previously published data (*r*^2^ = 0.98, Additional files 12 and 13). Thus, we concluded that this method could be used to study RNA editing in detail in the developing brain.

### Different brain regions have different levels of Adar transcripts during development

**Fig. 2.**
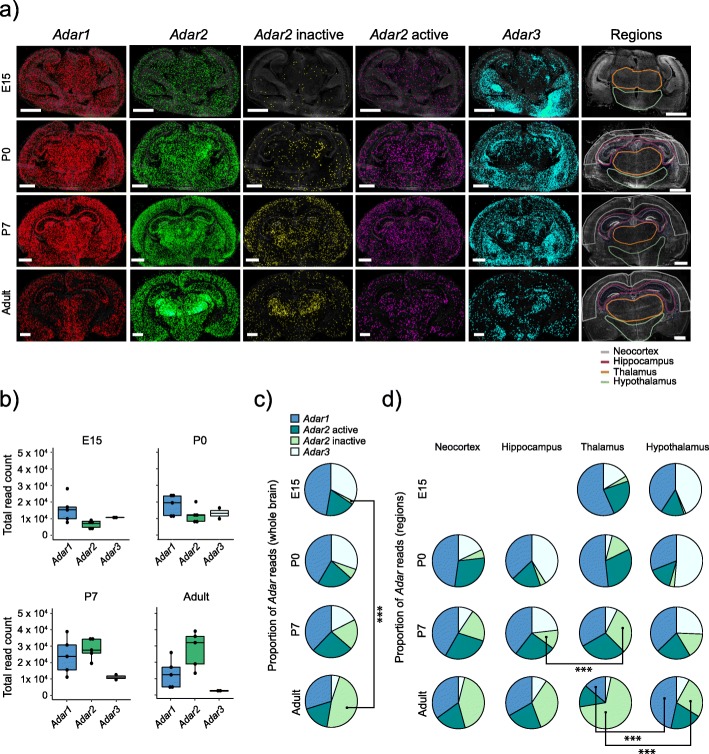
Relative expression of *Adar1*, *Adar2,* and *Adar3* transcripts. **a** The spatial distribution of *Adar1* (red), *Adar2* (green), *Adar2* inactive (yellow), *Adar2* active (purple), and *Adar3* (cyan) transcripts and the regional selection outlined for each developmental stage. The scale bar is 1 mm. **b** Total number of reads for *Adar1*, *Adar2,* and *Adar3* for each developmental stage (Additional files 7, 8, 9, and 10), each replicate is represented by a dot. As different developmental stages have different levels of background autofluorescence, resulting in varying quality thresholds for optimal read quality, the read counts in adults are lower than expected. An increase in expression of *Adar2* was observed from E15 to adult by non-parametric Kruskal-Wallis (*p* = 8.7E−4). **c** The relative proportion of reads, an average based on the replicates from each developmental stage, for *Adar1*, *Adar2,* and *Adar3* showing a developmental increase in the proportion of the inactive *Adar2* splice isoform. The proportions of active and inactive *Adar2* transcripts are based on the derived level of auto-editing of *Adar2*. **d** Regional read proportions of *Adar1*, *Adar2,* and *Adar3*, presented as in **c**. Hippocampus and neocortex could not be outlined for E15 and hence only data for thalamus and hippocampus are presented for this stage. Asterisks indicate the level of significance for the observed differences, *** *p* < 0.001

To analyze the temporal as well as the regional expression of the RNA-editing enzymes *Adar1*, *Adar2*, and *Adar3*, we extracted data from neocortex, hippocampus, thalamus, and hypothalamus and from the whole brain for the transcripts encoding all three members of the *Adar* family. Five replicates were analyzed for expression of *Adar1* and *Adar2*, while two replicates were analyzed for *Adar3* expression. Our analysis of the developmental and the regional expression revealed that the relative expression of these transcripts varied during brain development. The levels of expression of *Adar1*, *Adar2*, and *Adar3* were initially similar but the level of *Adar2* increased during development to become the dominant *Adar* transcript in the adult brain (*p* = 0.00087, non-parametric Kruskal-Wallis) (Fig. 2a, b). The expression of *Adar1* and *Adar3* remained fairly constant during development (non-parametric Kruskal-Wallis with Dunn-Sidak was non-significant). PLPs were used to detect the two splice variants of *Adar2* to determine if the level of *Adar2* auto-editing was constant in different brain regions during development. We confirmed previously published reports that *Adar2* mRNA expression increases during brain development [21]. This increase is caused by an increase in the transcript encoding the inactivated protein form, a consequence of auto-editing, while the expression of the active form remained unchanged (Fig. 2c). As an increase in generating *Adar2* inactive transcripts is due to auto-editing, these results indicated a developmental increase in ADAR2 activity. The protein levels of the Adar enzymes were not investigated.

The relative expression of *Adar1*, *Adar2*, and *Adar3* also varied within the examined brain regions (Fig. 2a, d). As we were interested in how the relative levels of the transcripts of the Adar family were changing during development and how they affected the general editing level, we compared the regional proportions of the different transcripts. The regional expression profiles of the transcripts encoding the members of the *Adar* family were most distinct in the adult brain (Fig. 2d and Additional file 14), but already in P7 there was a larger proportion of the inactivated *Adar2* transcript variant in thalamus. Moreover, the proportion of the auto-edited inactive variant of *Adar2* in this region indicated that the activity of ADAR2 was higher in this region than in the other regions. We observed a trend of a higher proportion of *Adar3* (*n* = 2, significance not shown) in hypothalamus in comparison to thalamus in E15, and similarly in P0, where hippocampus also had a relatively high proportion of *Adar3* compared to thalamus and neocortex. The observed proportional difference in *Adar1* expression between thalamus and hypothalamus in adult brain was likely an effect of the high level of expression of *Adar2* in thalamus as the observed numbers of *Adar1* reads did not differ between the developmental stages. These results showed that the expression level of transcripts encoding the different members of the *Adar* family varied during development as well as between the examined regions. The regional level of auto-editing indicated that the activity of the ADAR2 enzyme also differed between the examined regions.

### Thalamus exhibits increased editing activity

**Fig. 3.**
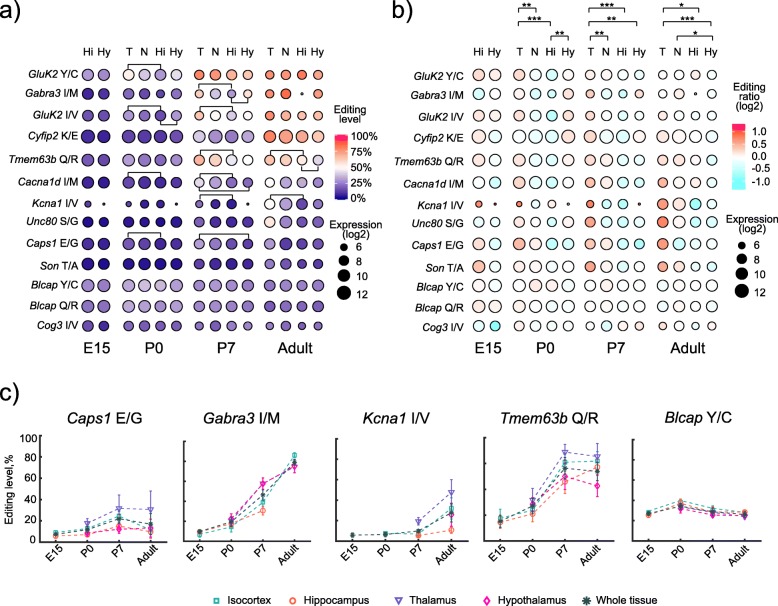
Regional editing. **a** Bubble charts of the editing level (derived from data in Additional files 7, 8, 9, and 10) for the investigated editing sites, by developmental stage, for the regions thalamus (T), neocortex (N), hippocampus (Hi), and hypothalamus (Hy). The color of the bubble indicates the average level of editing and the size of the bubble the average level of expression (log2 of the number of reads) within that particular region. Lines connecting bubbles indicate regions where the editing levels differ based on statistical testing with Kruskal-Wallis and post hoc Dunn-Sidak. The level of significance is presented in Additional file 9. **b** Bubble charts of the editing ratio (regional editing level for an editing site over the editing level of that editing site for the whole brain with the region excluded) for the investigated edited sites, by developmental stage, where the color indicates if the regional editing level is elevated (red), similar (white) to or lower than (cyan) the outside area. The stronger the color, the larger the deviation in editing level. The level of difference (Dunn pairwise testing with correction for multiple testing) between the regions are indicated as *p < 0.05, **p < 0.01, and ***p < 0.001 at the top of the charts and also presented in Additional file 8: Table S6. NA values (due to low read count) in gray. **c** The spatiotemporal editing level for *Caps1* E/G, *Gabra3* I/M, *Kcna1* I/V, and *Tmem63b* Q/R, editing sites which display regionally different editing levels. As a reference, *Blcap* Y/C shows no regional deviations in the editing level. Data points are missing for neocortex and hippocampus in E15, as these regions could not be outlined at this stage of development. Other missing data points have been excluded due to low read count

To investigate how the editing levels varied between the selected brain regions and their relation to the regional expression of the transcripts of the enzymes of the *Adar* family, the regional editing levels for each investigated site were derived from the included replicates (exact number, 5 or 2, stated in Additional file 1) and visualized as replicate average values for the different developmental stages (Fig. 3a). As expected, a temporal increase in general editing activity was observed. However, this temporal increase in editing activity differed between the selected brain regions. To investigate whether the regional editing activity was higher or lower than in other regions, a ratio of regional editing level over the editing level of the area outside the region was calculated for each editing site. The editing ratio for each editing site in each replicate was calculated based on the editing level within a selected ROI and the editing level for that editing site in the whole brain excluding the ROI. The regional editing ratios for all editing sites (mean editing ratio for included replicates per developmental stage) were visualized and compared, revealing that there was generally higher editing activity in the thalamic region from P0 and later in development, as more transcripts were edited to a higher level in comparison to the other regions (Fig. 3b and Additional file 15). The regional expression of *Adar* transcripts and the observed regional editing activities indicated that different ADAR proteins could be responsible for the regional differences in editing activities which varied with the developmental stage. In P7 and adult tissues, high editing in the thalamus of the known ADAR2 substrates *Kcna1* I/V [30] and *Cyfip2* K/E [31] and auto-editing of *Adar2* [4] indicated high activity of ADAR2 in this region (Figs. 2d and 3b). Based on the enrichment of the edited *Unc80* S/G transcript in thalamus in adult brain, our data suggested that it may be an ADAR2-specific substrate. This was further supported by high co-expression of *Adar2* and the *Unc80* edited transcript at a cellular level (Additional file 16). In contrast, no regional co-localization was observed for *Adar1* and its substrates *Blcap* Y/C and Q/R, indicating that *ADAR1* editing was more constitutive and ubiquitous and less likely to contribute to variations in editing that are region-specific (Additional file 14). Interestingly, the regional variation in editing activity at the early stages E15 and P0 did not correlate well with either *Adar1* or *Adar2* expression. Instead, we saw indications (*n* = 2, no significance shown) that early in development the higher editing level in the thalamic region correlated better with the low level of *Adar3* expression, while the lower editing activity in hypothalamus correlated with the higher expression levels of *Adar3* (Fig. 2a). These results suggested that regional differences in editing in early development may be driven by ADAR3, acting as an inhibitor of editing [10].

To further analyze the data and find the individual transcripts whose editing contributed to the regional editing profiles, we compared the regional editing levels of individual transcripts. The editing levels for the analyzed editing sites were derived from either five or two replicates (indicated in Additional file 1). For a number of editing sites, we found that their respective editing level differed between the analyzed regions (Fig. 3a and Additional file 17). Regional differences could only be shown with confidence for editing sites covered by five replicates. We observed that regional editing profiles emerged in P0 and that differences mostly existed between thalamus and hippocampus, with lower editing levels in the latter. For *Caps1* E/G, the editing level was higher in thalamus than in hypothalamus in P7 (Fig. 3c). *Gabra3* I/M was on the other hand more highly edited in hypothalamus than in hippocampus at the same developmental stage (Fig. 3c). In adult brain, editing of the Q/R site in *Tmem63b* was higher in both neocortex and thalamus compared to hypothalamus (Fig. 3c). Editing of *Kcna1* I/V was also higher in thalamus than in hippocampus in adult brain (Fig. 3c). In contrast to the edited sites for which we observed regional differences in editing level, for other sites, such as the Y/C site of *Blcap*, no regional editing profile was observed (Fig. 3c).

Thus, this spatial analysis revealed that regional differences in the level of editing emerge already at P0 and mainly in a specific subset of edited transcripts.

### Single-cell analysis reveals a developmentally increasing complexity in the regulation of editing at a subset of editing sites

Variation in levels of RNA editing of transcripts coding for proteins involved in neurotransmission has been suggested to contribute to functional diversity in the brain [6]. Previous studies have also indicated that different cell types in the brain are characterized by specific editing patterns, though cell-type-specific patterns in editing of recoding sites has not been specifically addressed [25]. Furthermore, how these patterns emerge during development remains to be shown. We took advantage of the single-cell resolution of our method and analyzed the editing levels of our selected sites in cells that were also positive for cell type markers for interneurons (*Cck*, *Ndnf*, *Nrn1*, *Pvalb*, *Sst* and *Vip*), pyramidal neurons (*Pcp4*), oligodendrocytes (*Plp1* and *Enpp2*), and astrocytes (*Gfap*). In summary, single cells were segmented based on a fixed distance from the DAPI-stained nuclei, and reads detected within these borders were assigned to that cell (for details, see “Methods” and Additional file 18). Next, editing levels for the different transcripts were calculated for the populations of cells that were positive for a particular cell type marker (Additional file 19). Finally, the editing levels of marker-positive cells for each individual editing site were compared to the editing levels associated with the other markers. The ratio of marker-positive editing level over the marker-negative was also calculated to investigate whether the marker-associated editing level was higher or lower in comparison to cells where the marker was not detected. We first compared the general editing profile, in the form of editing ratios, associated with each of the included markers to assess the cell-type-specific editing activity (Additional file 19). At each developmental stage, we found a higher editing activity in cells positive for markers of interneurons and pyramidal neurons in comparison to cells positive for markers for astrocytes (*Gfap*) or oligodendrocyte (*Enpp2* and *Plp1*) (Table 1). Four individual transcripts (*Cacna1d*, *GluK2*, *Kcna1*, and *Tmem63b*) showed cell type-specific regulation of RNA editing by this analysis (Table 2). These results revealed that A-to-I editing of individual editing sites can be specifically tuned in certain cell types.

**Table 1:** Cell-type-specific editing profiles. The markers where pairwise testing identified a difference in editing profile

Developmental stage	*p* (Dunn-Sidak)	Editing level	
		High	Low
E15	0.0350	Sst	Enpp2
P0	0.0002	Nrn1	Gfap
	0.0337	Sst	Gfap
	0.0219	Pcp4	Gfap
P7	0.00022	Nrn1	Gfap
	0.00013	Pvalb	Gfap
	0.01842	Pcp4	Gfap
Adult	0.0210	Pvalb	Plp1

**Table 2:** Cell-type-specific editing of individual sites. The individual sites which were found to be edited to different extent in cells positive for different markers

Developmental stage	Edited site	*p* (Dunn-Sidak)	Editing level	
			High	Low
P0	Gluk2 Y/C	0.028	Nrn1	Gfap
P7	Cacna1d I/M	0.0135	Nrn1	Gfap
	Cacna1d I/M	0.0087	Pvalb	Gfap
	Gluk2 I/V	0.0039	Pvalb	Gfap
Adult	Cacna1d I/M	0.049	Nrn1	Gfap
	Kcna1 I/V	0.021	Pvalb	Plp1
	Tmem63b Q/R	0.039	Cck	Gfap
	Tmem63b Q/R	0.0033	Pvalb	Gfap
	Tmem63b Q/R	0.012	Pvalb	Plp1

**Fig. 4.**
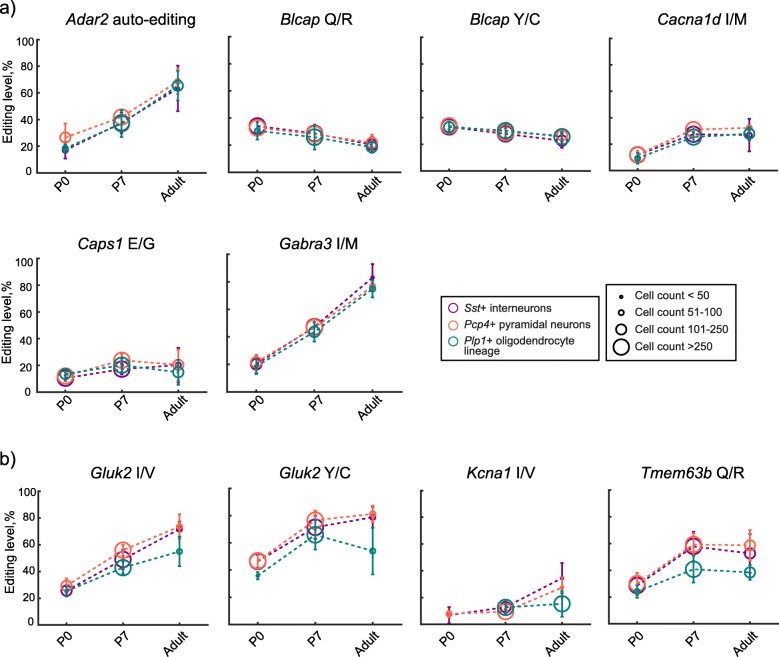
Neuronal and oligodendrocyte-associated editing. The temporal editing level in *Sst*-positive interneurons (purple), *Pcp4*-positive pyramidal neurons (red), and *Plp1*-positive oligodendrocyte lineage (green) for editing sites that did not show any difference in editing level between the three cell classes in **a** and for the editing sites where there were differences in editing level in **b**. The discovered differences (by Kruskal-Wallis testing followed by post hoc Dunn-Sidak) and the level of significance are presented in Additional file 13. The marker size of each data point indicates the number of cells (mean number for all replicates) upon which the data is based

Hwang et al. observed that the increasing editing level during brain development was temporally associated with neuronal maturation [26]. Furthermore, increased editing has been observed in primary cultures of mouse neurons as they mature in vitro [18]. These results suggest that the developmental increase in editing we observed could be caused by neuronal maturation as mature neurons are characterized by higher levels of editing. To investigate this possibility, we classified cells as *Sst*-positive interneurons, *Pcp4*-positive pyramidal neurons, and *Plp1*-positive oligodendrocyte lineage and analyzed editing levels for these three cell types. We found an increase in the editing of transcripts in both types of neurons, as well as in the oligodendrocytes, indicating that neuronal maturation is not the sole cause of the increasing editing (Fig. 4a). However, for *Kcna1* I/V, *Tmem63b* Q/R, *GluK2* I/V, and Y/C, the increase in editing was more prominent in neuronal (positive for *Sst* or *Pcp4*) cells and in general editing levels were lower in oligodendrocytes in P7 and in the adult brain (Fig. 4b and Additional file 20). This may be due to an observed lower expression of *Adar1* and *Adar2* in the *Plp1*-positive cells in the adult brain compared to cells expressing the *Sst* and *Pcp4* (Additional file 19), but also due to a precise regulation of the editing of certain sites, depending on the cell type where they are expressed.

**Fig. 5.**
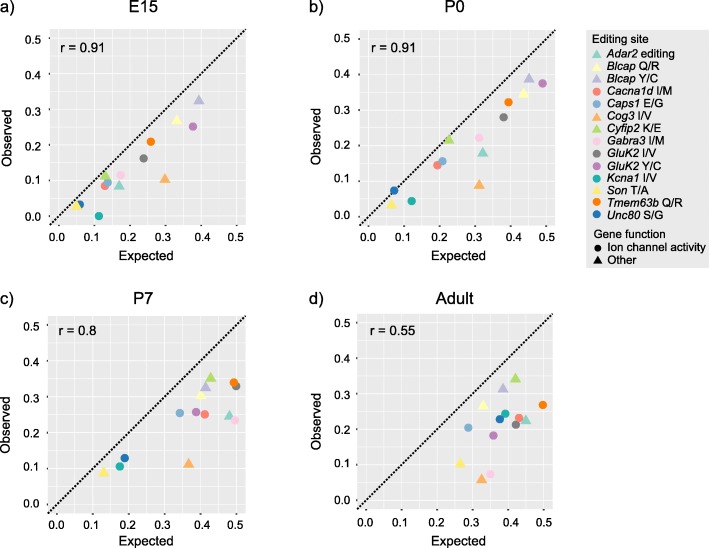
Single-cell data. Dot plots showing the correlation between observed and expected cells with both edited and unedited transcripts (mixed cells) for the developmental stages E15 in **a**, P0 in **b**, P7 in **c,** and adult in **d**. Each dot or triangle is one edited substrate, where the shape indicates the classification based on gene function (Additional file 21). The observed proportion of mixed cells is extracted from single-cell data for cells with at least one read each of the edited and the unedited substrate assigned. The expected proportion is calculated from the observed editing level, assuming a random distribution of reads. The dotted line indicates a perfect correlation and provides a point of reference for the observed under-representation of mixed cells compared to what would be expected

Observed editing levels in bulk tissues have been shown in many cases to result from heterogeneity in editing levels among individual cells and require single-cell-level analysis [32]. By studying the population of cells with at least two assigned reads of a particular transcript in our data, we sought to examine the RNA editing at the single-cell level. The single cells were defined as described above (also, see “Methods” and Additional file 18). We compared the observed number of cells with both edited and unedited reads (referred to as mixed cells) to the expected frequency by Pearson correlation, assuming a random distribution. We found that the observed frequency of mixed cells matched a random distribution early in development, in E15 and P0. However, later in development, an under-representation of mixed cells for an increasing number of edited sites was observed (Fig. 5). Interestingly, at later stages of development, some of the transcripts matched the expected frequency for a random distribution, while most did not. To assess if there was any functional dimension to the observed patterns of expected and observed mixed cells, the transcripts of the edited sites were categorized based on their associated Gene Ontology terms, as involved in “Ion channel activity” or as “Other” (Additional file 21). All the edited transcripts that were classified as involved in ion channel activity, except for one (*Caps1*) that is a regulator of dense core vesicle exocytosis [33], turned out to be those that deviated from the expected frequency of mixed cells in the adult mouse brain. This category of transcripts appeared to exhibit more bimodal distributions of editing levels among cells expressing the transcript (Fig. 5), as in more cells than expected by chance are positive for signals of the same kind, edited or unedited, for a certain transcript. These transcripts were also those found to be differentially edited in the examined regions and/or in different cell types (Table 2 or Additional file 17).

These results showed, and further support, our previous observations that there is an increasing complexity in the regulation of editing of specific transcripts during development, resulting in significant cell-to-cell variation in the adult brain. The function of the proteins encoded by the analyzed transcripts points toward that this increasing level of complexity may be associated with the regulation of editing of transcripts likely involved in different aspects of neurotransmission.

## Discussion

While it is likely that a range of known and unknown trans-regulators of RNA editing play a role in shaping the RNA-editing landscape observed in this study, some of the variation in editing is the result of differential expression of the ADAR proteins. Furthermore, ADAR2 is able to edit its own transcript, leading to a frameshift and downregulation of the levels of the active enzyme. We confirmed previous observations that while the *Adar2* mRNA expression increases during brain development, the active form of the transcript remains at a fairly constant level [21] most likely reflecting the increased activity of the ADAR2 protein during brain development, though it is possible other unknown mechanisms underlie the increase in detected levels of the auto-edited form of ADAR2 mRNA. Previous publications have suggested mechanisms for this developmental increase in ADAR2 activity, including an increased import into the nucleus and increased protein stability [18].

Across the developmental stages, we observe regional differences in editing activity that can be partially accounted for by regionally varying proportions of *Adar1*, *Adar2,* and *Adar3* transcripts. This indicates fundamentally differing roles for the three ADAR proteins in shaping the brain editome, with fairly ubiquitous ADAR1 expression providing a “baseline” level of editing throughout the brain, while differential ADAR2 and ADAR3 expression modulates editing variations across different regions. Furthermore, our results points to that ADAR3 may act as an important regulator of RNA editing in early brain development. The functional significance of the high levels of RNA editing and ADAR2 activity in the thalamus remains unknown but could reflect the contribution of RNA editing to neuronal diversity particularly important in this region of the brain.

During brain development, editing levels of the studied RNA substrates were either stable or increasing [21, 26], which may be associated with neuronal maturation and also underlies the developmental increase [26]. Editing levels are generally higher in neurons than in oligodendrocytes, but the developmental increase in editing is also occurring in oligodendrocytes, indicating that neurons are not the sole source of the increasing editing pattern. As previously mentioned, the activity of ADAR2 is known to be promoted by at least two other proteins: importin-a4 (encoded by *Kpna3*) facilitates ADAR2 nuclear import [18] and Pin1 acts as a stabilizer of ADAR2 in the nucleus [17]. The online database mousebrain.org contains single-cell RNA sequencing data for the mouse brain and allows for searching for combinatorial gene expression patterns [34]. A database search for *Adar1* (*Adar*), *Adar2* (*Adarb1*), *Kpna3,* and *Pin1* shows that the expression of the four transcripts is relatively high in both excitatory and inhibitory neurons in cerebral cortex and thalamus but also in some cells in hypothalamus (data not shown). Meanwhile, non-neurons, such as oligodendrocytes and astrocytes, show a much lower expression of all four of the transcripts. As we could observe similar editing levels for some of the studied transcripts in interneurons and pyramidal neurons compared with oligodendrocytes, this suggests that editing level does not simply correlate with the expression of the editing enzyme. This presents exciting possibilities to identify novel trans-regulators of RNA editing. Future studies could identify interactors of the substrate in regions with aberrant editing to explain observations such as this.

The six transcripts whose editing level differs between regions and/or between different cell types can all be associated to functions related to ion channel activity or other aspects of neurotransmission (Additional file 21). Editing of these substrates has been implicated to have an impact on functions such as regulation of the surface expression of neuro-receptors (*Gabra3* and *Kcna1*) [35, 36], of neurotransmission (*GluK2* and *Kcna1*) [30, 37], and of signal transduction (*Cacna1d*) [38]. *Caps1* (also called *Cadps*) is a Ca^2+^-dependent activator of exocytosis of dense core vesicles [33]. *Tmem63b* is not well-studied but recent results suggest a function as an osmosensitive Ca^2+^ channel [39]. In contrast, the four transcripts, for which we could not see any region-specific editing profiles, could not be associated with any functions related to cell-to-cell signaling or ion transport (Additional file 21). This suggests that the regulation of specific editing is of particular importance in transcripts associated to cell-to-cell signaling and ion channel activity, likely contributing to diversity of neuronal signaling.

In this study, we performed an analysis of single-cell-level editing heterogeneity by determining the frequency of cells expressing both edited and unedited isoforms of the analyzed substrates (mixed cells). The developmental decrease in mixed cells, compared to a random distribution, indicates that in the adult brain, the editing levels observed by bulk tissue RNA sequencing reflects the average editing level of cell populations with divergent editing levels, reinforcing the necessity of single-cell resolution methods. Generally, observed heterogeneity in editing levels on a single-cell level can have two mechanistic explanations: a result of cell-intrinsic gene expression of editing regulators or a result of external signals and of histological context. Additionally, this analysis revealed that single-cell editing level heterogeneity arises at the later stages of development and only for certain substrates. Our analyzed developmental stages cover a period where the brain undergoes a dramatic increase in complexity, with extensive neuronal maturation, migration, and the formation of a mature synaptic network. The emergence of editing level diversity during this process supports the idea that editing can contribute to the complexity of the brain. Furthermore, this interpretation can support a division of editing substrates into those that contribute to neural complexity, often via modulation of neurotransmission, and those that do not and instead represent “housekeeping” substrates that are more constant in editing level.

With this method, we have analyzed the RNA editing profile of hundreds of cells classified by expression of certain markers or by their tissue location. This provides an overview of spatial and cell-type-specific editing signatures. The brain is a large and complex organ, for which we have provided snapshots in time for a limited region. We were able to reveal regionally different, as well as specific marker-associated RNA editing profiles, indicating that there are intricate regulatory mechanisms behind RNA editing. This further strengthens the notion that the purpose of RNA editing is to shape and fine-tune the functions of the different regions of the brain as well as that of single cells.

## Conclusions

Our study reveals a developmentally increasing level of diversity in editing through regional as well as cell-type-specific regulation, features that would not be discovered through bulk analysis. These discoveries provide exciting new directions for studies of A-to-I RNA editing on a single-cell level to further study the different layers of regulation of RNA editing, with future studies possibly expanding the list of analyzed editing sites to include additional recoding editing sites and sites in non-coding RNA such as microRNAs.

Furthermore, editing has been shown to contribute to cancer immunotherapy resistance [40], and a large amount of editing sites have been found to be differentially edited in several cancer types [41] and this method provides a novel way to study the development of dysregulation of editing during tumor progression.

## Methods

### Preparation of brain tissue sections

Whole brain tissue were prepared from NMRI mice (Charles River) at the developmental stages E15, P0, P7, and adult. All brains were collected in PBS on ice. After two washes in DEPC-PBS, the tissues were embedded in OCT (Thermo Fisher Scientific) and instantly placed on dry-ice before they were stored at − 80 °C. Subsequently, 10-μm coronal tissue sections from the brains (three tissues for each stage) were prepared on a cryostat (Becton Dickinson (BD)). Tissue sections were then stored at − 80 °C until use.

### Padlock probe design

The padlock probes targeting the editing sites were designed to hybridize 18–20 nucleotides downstream and 18–20 nucleotides upstream of the editing site with the probe’s 3′ nucleotide being the nucleotide interrogating the edited site (A or G). Interspacing the target-specific sequences was a linker sequence containing recognition sites for an anchor primer as well as the interrogation probes. The linker sequence also contained a four nucleotide barcode, unique for each of the targets included in the panel. For the markers and the Adar transcripts where we were not limited to a particular site, a set of probes was designed for each of them to maximize the number of reads obtained. Each set targeting a specific transcript was different in the target-specific sequence, but identical in the linker sequence with one barcode unique for that particular target.

### In situ reverse transcription, barcode padlock probing, and rolling circle amplification (RCA)

For the tissue sections, fixation was performed in 3.7% (w/v) paraformaldehyde (Sigma) in DEPC-treated PBS with 0.05% Tween-20 (Sigma) (DEPC-PBS-T) for 45 min at RT and two washes in DEPC-PBS-T. The tissue was permeabilized in 2 mg/ml pepsin (Sigma) in 0.1 M HCl at 37 °C for 2 min. After two washes in DEPC-treated PBS and dehydrated in an ethanol series, tissues on the slide were then covered with Secure-Seal hybridization chambers (Invitrogen) for the following experiment. For cell line, Secure-Seal hybridization chambers were used directly on a cell slide after an ethanol series of 70%, 85%, and 100% for 1 min each to remove water. Both cells and tissue samples were first rinsed with DEPC-PBS-T. A reversed transcription mix, containing 5 μM of unmodified random decamers, 0.2 μg/μl BSA (NEB), 500 μM dNTPs (Fermentas), 20 U/μl of TranscriptMe reverse transcriptase (Gdansk), and 1 U/μl RiboLock RNase Inhibitor (Fermentas) in the TranscriptMe reaction buffer, was applied on the slides. The sequences of all oligonucleotides used in this study are listed in Table S2. The reverse transcription was carried out for 3 h at 37 °C for the cell slides and overnight for the tissue sections. Slides were then washed twice with DEPC-PBS-T, and followed by a post-fixation step in 3.7% (w/v) paraformaldehyde in DEPC-PBS for 30 min at room temperature. After post-fixation, the samples were washed twice in DEPC-PBS-T. After the reverse transcription, RNA degradation, hybridization, and ligation of padlock probes on synthesized cDNA were performed. A mix which contained 1× Ampligase buffer (20 mM Tris-HCl, pH 8.3, 25 mM KCl, 10 mM MgCl2, 0.5 mM NAD, and 0.01% Triton X-100), 100 nM of each padlock probe, 50 μM dNTPs, 0.5 U/μl Ampligase, 0.4 U/μl RNase H (Fermentas), 50 mM KCl, and 20% formamide was added to the sample reaction chambers on slides. The slides were incubated at 37 °C for 30 min, and 45 °C for 45 min, and then washed twice with 1× DEPC-PBS-T. For the RCA, the slides were incubated with a RCA mix (1 U/μl phi29 polymerase (Fermentas), 1× phi29 polymerase buffer, 0.25 mM dNTPs, 0.2 μg/μl BSA, and 5% glycerol in DEPC-H2O) for 2.5 h. For tissue slide, RCA was carried out overnight, followed by washing two times with 1× DEPC-PBS-T.

For technical validation of the use of padlock probes in situ, we refer to the original publications of the method [28, 42].

### Sequencing by ligation, and image acquisition

Sequencing of the barcodes of the RCA products (RCPs) was carried out by applying a hybridization mix containing 500 nM of anchor primer in 2× SSC and 20% formamide to the sample followed by incubation at RT for 30 min and subsequent washing two times with DEPC-PBS-T. A ligation mix containing each interrogation probe, 100 ng/ml of DAPI, 1 mM ATP (Fermentas), 1× T4 ligase buffer (Fermentas), and 0.1 U/μl of T4 ligase (Fermentas) was added to the samples and incubated for 30 min at RT. After washing three times with DEPC-PBS-T, the slide was mounted in SlowFade Gold Antifade Mounting Media (Thermo Fisher Scientific). In the following rounds of sequencing, an ethanol series was first used to remove the mounting medium from the tissue section. Slides were washed with DEPC-PBS-T once and treated with UNG buffer (1× UNG buffer (Fermentas), 0.2 μg/μl BSA, 0.02 U/μl UNG (Fermentas)) for 30 min at 37 °C and washed twice with DEPC-PBS-T before washing three times with 65% formamide for 60 s each to strip off the previous interrogation probes. The hybridization of anchor primer or interrogation probes was then performed similarly as the previous round.

Images were acquired using an Axioplan II epifluorescence microscope (× 20 objective). Exposure times for all the experiments are listed in Additional file 22. After imaging, the slides were prepared for the next three sequencing cycles by UNG treatment buffer as described above followed by repeating the hybridization, ligation, and imaging. The images were acquired, as previously described [27], as a stack of images at different focal depths and then merged to maximum intensity projection image followed by stitching using the Zeiss AxioVision software Zen.

### Image analysis

The automated sequence decoding was performed as previously described [27]. In short, cell nuclei were separated based on shape descriptors (Additional file 18). The definition of cell cytoplasm uses the nucleus as a seed and sets the cell border 20 pixels from the nuclei border. Borders between cells in close proximity were drawn based on watershed segmentation. Thus, there are no overlapping cells. The image of the general stain was enhanced by a top-hat filter, and RCPs were separated by watershed segmentation. The images were aligned, and fluorescence intensity from each of the signals representing A, C, T, and G were extracted. The optimal transformation between a merged image of all signals (A + C + T + G) and the general stain was determined based on intensity [43]. Analysis was performed with CellProfiler (2.1.1, 6c2d896) calling ImageJ plugins from Fiji for image registration. All intensity information was decoded using a script written in Matlab. Briefly, RCP was assigned for the base with the highest intensity for each RCP in all hybridization steps. A quality score was extracted from each base, and the quality of a called transcript was defined as the lowest quality of all the bases in the transcript barcode. The quality score ranges from 0.25 (poor quality) to 1 (good quality). The frequency of each sequence was extracted after a typical quality threshold of 0.35–0.5 was set. For the single-cell analysis, the cells located on tile edges during image acquisition were reanalyzed and the cell borders were adjusted in order to reduce the misalignment of cells due to the image stitching. The reads identified in cells on the tile borders were then assigned to the new edge-corrected cells based on original coordinates.

Furthermore, single-cell data was generated by assigning the reads to cells based on the coordinates of the read and segmented cells. Each defined cell is an approximation of a cell based on a fixed distance of 20 pixels from the identified nucleic border, and hence, our analyses are restricted to cell soma events.

### Data processing and analysis

Based on the in situ sequencing data, the mean editing level was calculated for each of the edited sites based on the number of reads for the edited and unedited variants of their respective transcript as was the expression level (the numbers of edited and unedited transcript reads combined). This was done based on whole brain data and based on data extracted from the selected regions neocortex, hippocampus, thalamus, and hypothalamus (Fig. 2a). The regions were manually selected as regions of interest (ROIs) in the microscopy images for each replicate. Data was extracted from each ROI in each replicate for further analysis. At E15, neocortex and hippocampus could not be identified, and hence, only thalamus and hypothalamus were analyzed for RNA editing at this stage. The outlining of the regions for P0, P7, and adult were based on Allen Brain Atlas while the region outlines in E15 brain sections was based on “The Atlas of Mouse Development” [29]. Expression and editing level were then visualized in bubble charts, where the color shows the editing level and the size of the bubble indicates the number of reads from which the editing level was calculated, mean values based on the biological replicates. Based on single-cell data, we also calculated the editing level and expression in populations of cells defined by their expression of the included cell type markers for the whole brain. Cells with a certain marker assigned to it were classified as positive and included, together with its assigned reads, in that marker class. We applied non-strict classification of cells meaning that in the event of multiple markers assigned to the same cell, that cell would belong to multiple cell classes. Mis-assignment of reads to the wrong cells would most likely be an effect of the cell segmentation strategy and be more probable with a higher expression level. For an edited target to be considered for analysis, at least 500 reads on average for the least prevalent of the unedited or edited transcript had to be detected at the whole tissue level for at least one developmental stage. This threshold was set to allow us to focus on transcript where we obtained a critical level of data. Target transcripts failing to fulfill this criterion were either lowly detected or showed generally very high or very low editing level with no temporal change. The analysis of regional Adar expression was based on the regional proportions of *Adar1*, *Adar2,* and *Adar3*. Based on the observed proportions of the two splice isoforms of *Adar2* resulting from intronic editing at the − 1 site, we estimated the proportions of the active and the inactive isoform based on the general read count for *Adar2*. These two isoforms were referred to as *Adar2* active (resulting from no editing at the − 1 site) and *Adar2* inactive (resulting from editing at the intronic − 1 site). The regional proportions for each Adar category were compared across each developmental stage by non-parametric Kruskal-Wallis with post hoc correction for multiple testing by Dunn-Sidàk. For the more detailed analyses of marker-specific and regional editing patterns, target transcripts falling below the set cutoff values in a particular region, cell type, or developmental stage were treated as NA values. For the analysis of marker-specific editing, at least 100 cells (average for all replicates of a developmental stage) expressing both the marker and the transcript being subjected to editing was required for further analysis. For the analysis of regional editing, a read count of 100 was required for a particular transcript (edited and unedited transcripts combined) for a replicate to contribute to the data for that transcript. In the analysis of temporal editing levels for *Sst*-positive interneurons, *Pcp4*-positive pyramidal neurons and *Plp1*-positive oligodendrocyte lineage, at least 20 cells were required per replicate. To test if the general editing level was differing between regions, we calculated the ratio of regional editing level over that of whole brain minus the region in question. In a similar way, marker-specific editing levels were compared as ratios of marker-specific editing level over the level registered in cells negative for that particular marker. The editing ratios were then visualized in bubble charts (Fig. 3b and Additional file 16: Figure S6 Marker-specific editing) and tested by the non-parametric Kruskal-Wallis test to identify differences in general editing activity. The Kruskal-Wallis test was followed by a post hoc Dunn-Sidak test (with correction for multiple testing) to identify the pairs of regions or markers that were differing. To find the individual transcripts who accounted for the observed differences between the regions and between the markers, the Kruskal-Wallis followed by Dunn-Sidak was applied to the editing levels obtained for each replicate for each individual edited site. Based on the regional and cell-type-specific editing profiles, the editing sites were classified as “regulated” and “unregulated”. Subsequently, a gene ontology analysis was performed of the two groups to investigate if they could be ascribed different general functions (Additional file 15).

## Additional files

Additional file 1: **Table S1**. Selected editing sites for the study. The editing sites (transcript followed by editing site) included in the target panel are listed together with their observed editing profile during development (Editing profile), the potential alternative transcript names (Alternative name), how they were processed in the analysis (Status), how many replicates the editing site was covered by and the reference number of the sequence used for the design of the padlock probes.

Additional file 2: **Table S2**. Padlock probes used for ISS. The padlock probes of the target panel and their respective sequence (5′ to 3′). Bold, underlined nucleotides at the 3′ end target the edited site, underlined nucleotides mark the position of the barcodes, nucleotides in bold are target-specific sequences and nucleotides in Italic are the sequences complementary to fluorescently labeled detection probes. The last column shows the barcode change made for the updated target panel.

Additional file 3: **Figure S1**. Spatial marker transcript distribution. The spatial expression of the included marker transcripts in adult brain tissue. The scale bar is 1 mm.

Additional file 4: **Figure S2**. Whole brain expression and editing. (a) The editing level for each edited site and each developmental stage. The color indicates the level of editing from low (blue) to high (red). (b) The same data as in (a), but after filtering based on read count for the edited and the unedited transcript variant (minimum 500 reads in the whole brain for the least prevalent variant). The grey color indicate NA values, meaning the edited sites and at which developmental stage that was filtered out. Edited sites with NA values for all developmental stages were not further processed. (c) Average expression data (reads/cell) for each edited transcript (reads for the edited and the unedited transcript variant combined) for each developmental stage. (d) Average expression (reads/cell) for the marker transcripts. The color indicates expression level ranging from low (blue) to high (red). Data derived from Additional files 7, 8, 9, 10.

Additional file 5: **Table S3**. Whole brain editing levels. The editing levels for the editing sites included in the analysis are shown as mean editing level for the included biological replicates for each developmental stage together with the standard deviation (SD).

Additional file 6: **Table S4**. Included brain tissue material. The brain sections processed in the study are listed together with the total number of cells segmented in each brain tissue and the average reads/cell for all detected targets. The ratio of expected reads indicates the quality filtering threshold set to obtain 95% or 96% expected reads out of total reads. The last column shows if the brain section was detected using the first or the second target panel of padlock probes.

Additional file 7: **Table S5**. Raw read counts E15. The raw read counts for the individual targets for each brain section and the selected regions after processing of the ISS image data.

Additional file 8: **Table S6**. Raw read counts P0. The raw read counts for the individual targets for each brain section and the selected regions after processing of the ISS image data.

Additional file 9: **Table S7**. Raw read counts P7. The raw read counts for the individual targets for each brain section and the selected regions after processing of the ISS image data.

Additional file 10: **Table S8**. Raw read counts adult. The raw read counts for the individual targets for each brain section and the selected regions after processing of the ISS image data.

Additional file 11: **Figure S3**. Replicate correlation. The correlation of ISS reads between the biological replicates from the same developmental stage.

Additional file 12: **Figure S4**. Correlation of observed editing levels to previously published editing levels. Also shown in Additional file 13.

Additional file 13: **Table S9**. Correlation of editing levels to published data. The editing sites included in our analysis are listed with their respective derived editing level for whole brain and editing levels found in published studies together with the respective reference [21, 33, 44–46].

Additional file 14: **Figure S5**. Regional Adar expression. The total number of reads per region for *Adar1*, *Adar2* and *Adar3* for each developmental stage (derived from Additional files 7, 8, 9, 10). Each dot represents a replicate. As different developmental stages have different levels of background autofluorescence, resulting in varying quality thresholds for optimal read quality, the read counts for adult are lower than expected. In E15, only the regions thalamus and hypothalamus are presented as neocortex and hippocampus could not be outlined at this stage.

Additional file 15: **Table S10**. Varying regional editing profiles. The differences in general editing profile found for the investigated regions for the different developmental stages together with the level of significance (Dunn-Sidak with correction for multiple testing). The region showing the lower or the higher editing activity in each comparison is also indicated.

Additional file 16: **Figure S6**. Adar-specific expression and editing. (a) Heat maps showing the Adar-specific expression (reads/cell) for each developmental stage, increasing age from left to right, derived from single cell data (data repository). The color indicates the level of expression from low (blue) to high (red). (b) Adar-specific editing for each developmental stage, presented in heat maps where the color indicates the editing level from low (blue) to high (red). The expression and editing have been calculated based on single cell data for the populations of cells which are positive for *Adar1*, *Adar2* or *Adar3* as well as for cells which are negative for all Adar transcripts. (c) Heat maps displaying the editing ratio for each developmental stage. The ratio is calculated as the Adar-specific editing level over the editing level in the *Adar* negative cells (negative for all Adars). The heatmap color indicates the level of under- (cyan) or over-editing (red). The stronger the color, the larger the deviation from the *Adar* negative cells.

Additional file 17: **Table S11**. Region-specific editing. The differences in regional editing found for individual editing sites at each developmental stage as well as between which regions the difference was found and at which level of significance (Dunn-Sidak with correction for multiple testing).

Additional file 18: **Figure S7**. Example of cell segmentation in adult brain. The blue lines mark the outlines of the cell nuclei, based on DAPI staining. The red lines mark the cell border, an approximation of the cell soma based on a fixed distance of 20 pixels from the nucleus border and watershed segmentation to separate cell nuclei in close proximity.

Additional file 19: **Figure S8**. Marker-specific editing. (a) Bubble charts displaying the editing level derived from the population of cells which are positive for a certain marker, based on single cell data (data repository). The color indicates the editing level. (b) Bubble charts of the marker-associated editing ratio, calculated as the editing level associated with a marker over the editing level of the cells negative for that particular marker. The color indicates if the marker-associated editing level is elevated (red), similar to (white) or lower than in other cells. The stronger the color, the larger the deviation. In both (a) and (b), the size of the bubbles indicates the size of the cell population (positive for both the marker and either of the edited or the unedited transcript variant). (c) The marker-specific expression of the ADAR transcripts. The color in the heatmap indicates the expression level of *Adar1*, *Adar2* or *Adar3* (log2) in cells positive for the respective markers. Data for marker-specific Adar expression where less than 20 cells were positive for both the marker and the respective Adar are indicated as NA values in grey.

Additional file 20: **Table S12**. Comparison of editing profiles for cells classified as *Sst*-positive interneurons, *Pcp4*-positive pyramidal neurons and *Plp1*-positive oligodendrocyte lineage at different developmental stages. The individual transcripts which were edited at different levels in the cell populations are listed together with the level of significance (Dunn-Sidak with correction for multiple testing).

Additional file 21: **Table S13**. Gene functions and editing categories. The gene functions found in literature and associated molecular processes from http://geneontology.org/ for the transcripts we analyzed and categorized by their spatial and cell-type-specific editing profile [33, 37, 39, 47–55]. The transcripts were classified as “Unregulated” when neither region-specific nor cell-type-specific editing could be found and “Regulated” when we observed differences in editing level between regions or between cell types. *Unc80* was categorized as “Potentially regulated” based on the spatiotemporal editing patterns, but due to insufficient data statistical significance could not be shown for the observed patterns.

Additional file 22: **Table S14**. Microscope exposure times (in milli-seconds) for the different channels used for ISS.

## Authors’ information

MÖ tragically deceased during the study and could not participate in the final writing of the manuscript.
