# Supplementary material for: Spatiotemporal mapping of RNA editing in the developing mouse brain using in situ sequencing reveals regional and cell-type-specific regulation
Source: BMC Biol. 2020 Jan 14;18:6. doi: 10.1186/s12915-019-0736-3 (PMC6961268; doi:10.1186/s12915-019-0736-3)

Figure S1. Spatial distribution of marker transcripts.

Interneuron

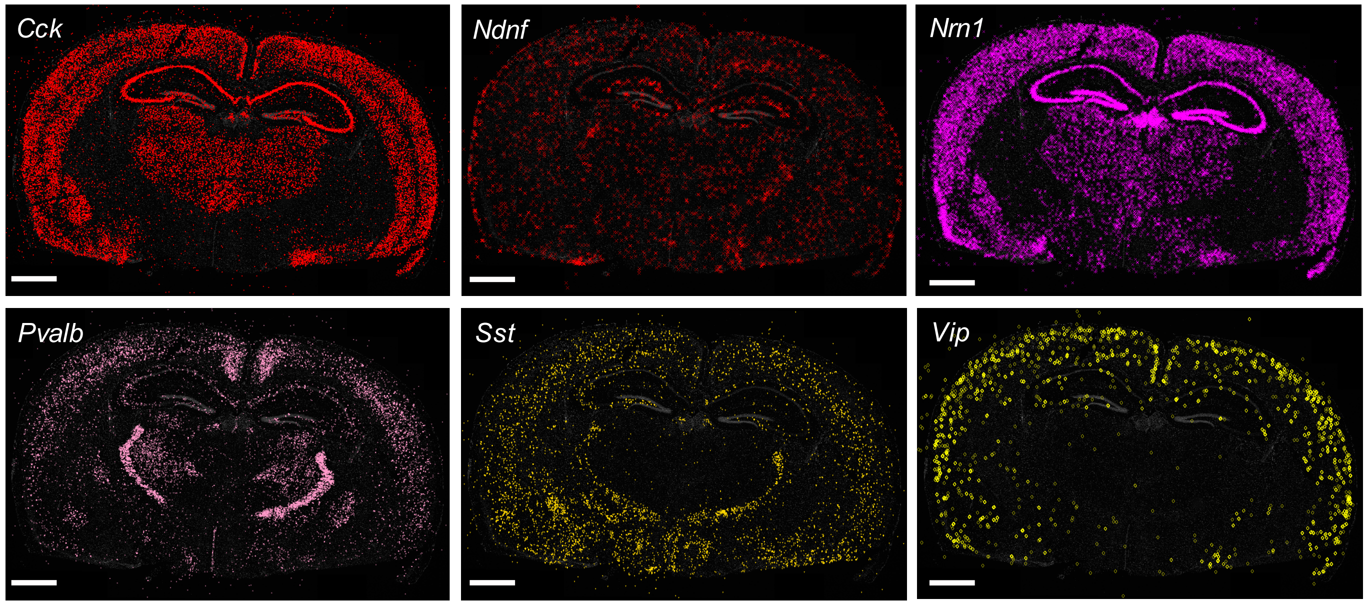

Pyramidal neuron

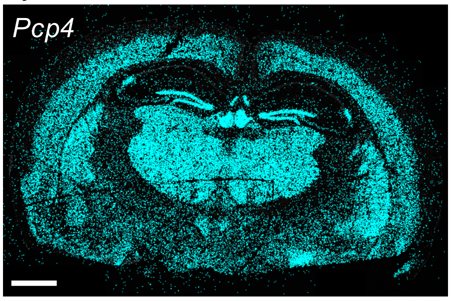

Oligodendrocyte

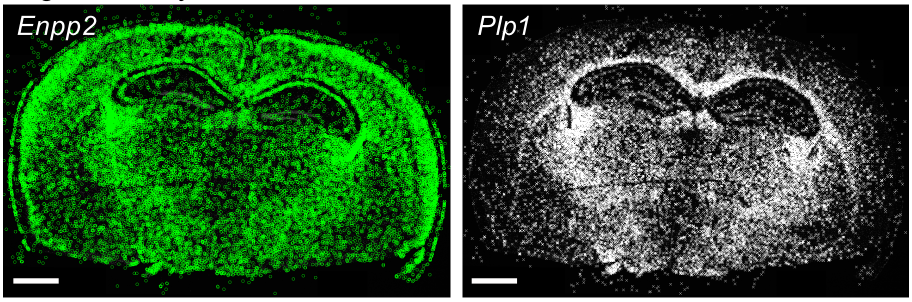

Astrocyte

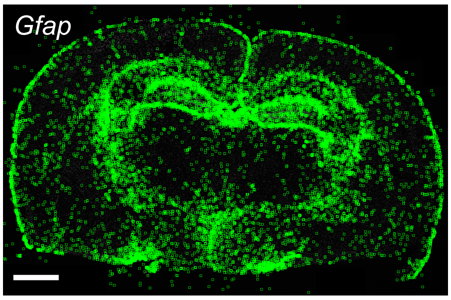

Supplement: Supplementary file 3 — Figure S1. Spatial marker transcript distribution. The spatial expression of the included marker transcripts in adult brain tissue. The scale bar is 1 mm. [file 12915_2019_736_MOESM3_ESM.pdf]
