# Supplementary figures and images for: Spatiotemporal mapping of RNA editing in the developing mouse brain using in situ sequencing reveals regional and cell-type-specific regulation
Source: BMC Biol. 2020 Jan 14;18:6. doi: 10.1186/s12915-019-0736-3 (PMC6961268; doi:10.1186/s12915-019-0736-3)

Figure S2. Whole brain expression and editing

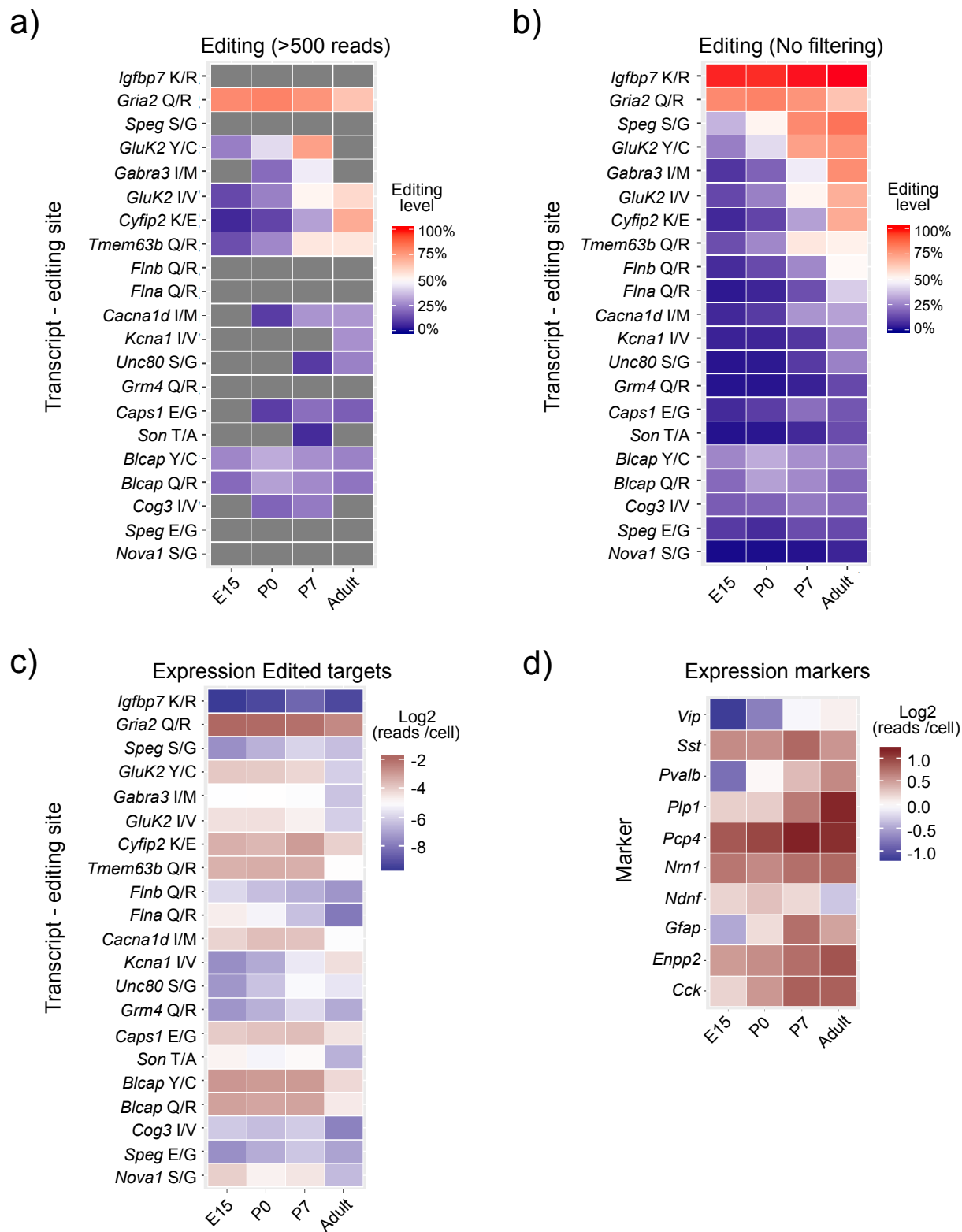

Supplement: Supplementary file 4 — Figure S2. Whole brain expression and editing. (a) The editing level for each edited site and each developmental stage. The color indicates the level of editing from low (blue) to high (red). (b) The same data as in (a), but after filtering based on read count for the edited and the unedited transcript variant (minimum 500 reads in the whole brain for the least prevalent variant). The grey color indicate NA values, meaning the edited sites and at which developmental stage that was filtered out. Edited sites with NA values for all developmental stages were not further processed. (c) Average expression data (reads/cell) for each edited transcript (reads for the edited and the unedited transcript variant combined) for each developmental stage. (d) Average expression (reads/cell) for the marker transcripts. The color indicates expression level ranging from low (blue) to high (red). Data derived from Additional files 7, 8, 9, 10. [file 12915_2019_736_MOESM4_ESM.pdf]

Figure S3. Intersample correlation.

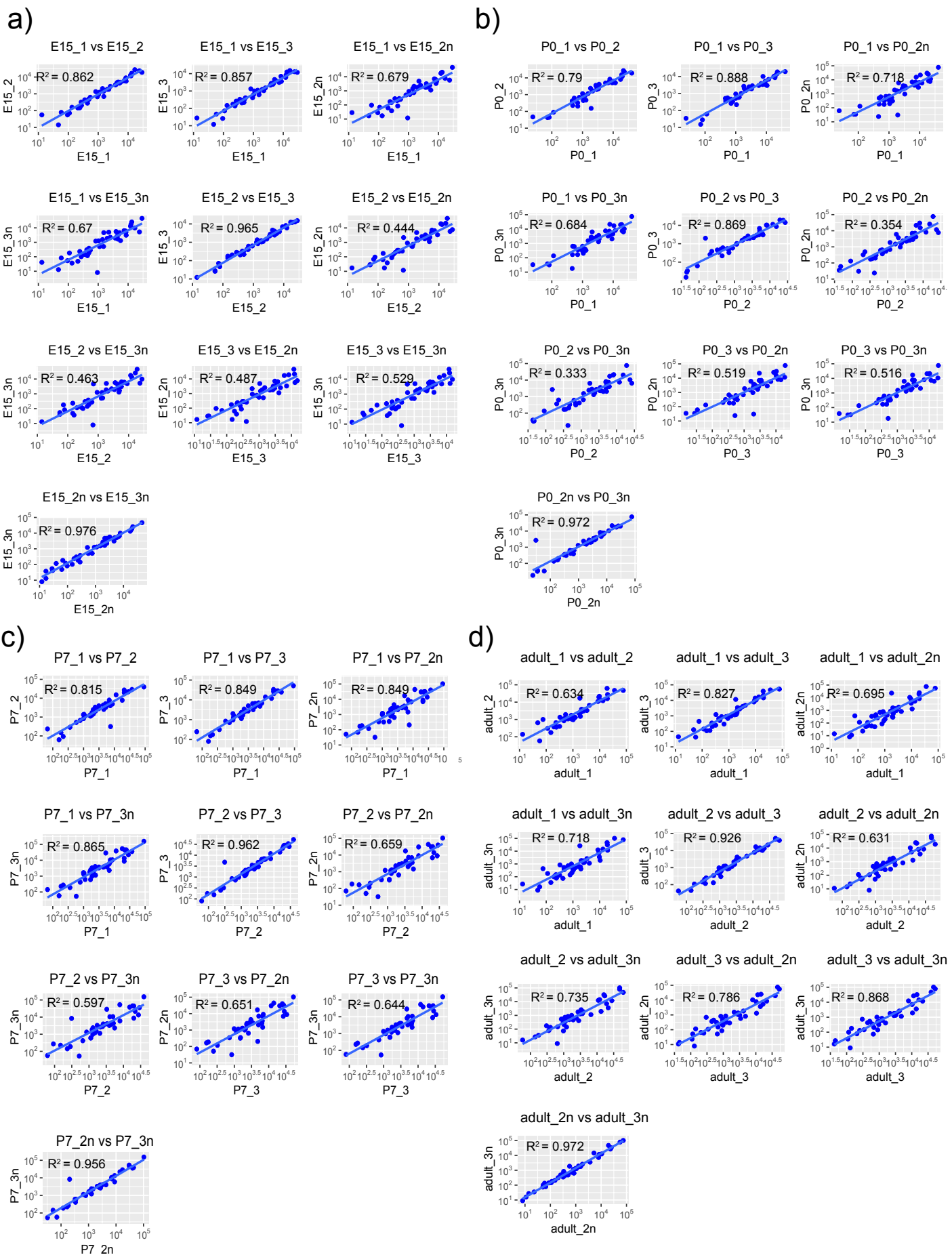

Supplement: Supplementary file 11 — Figure S3. Replicate correlation. The correlation of ISS reads between the biological replicates from the same developmental stage. [file 12915_2019_736_MOESM11_ESM.pdf]

Figure S4. Correlation of observed editing levels to published data

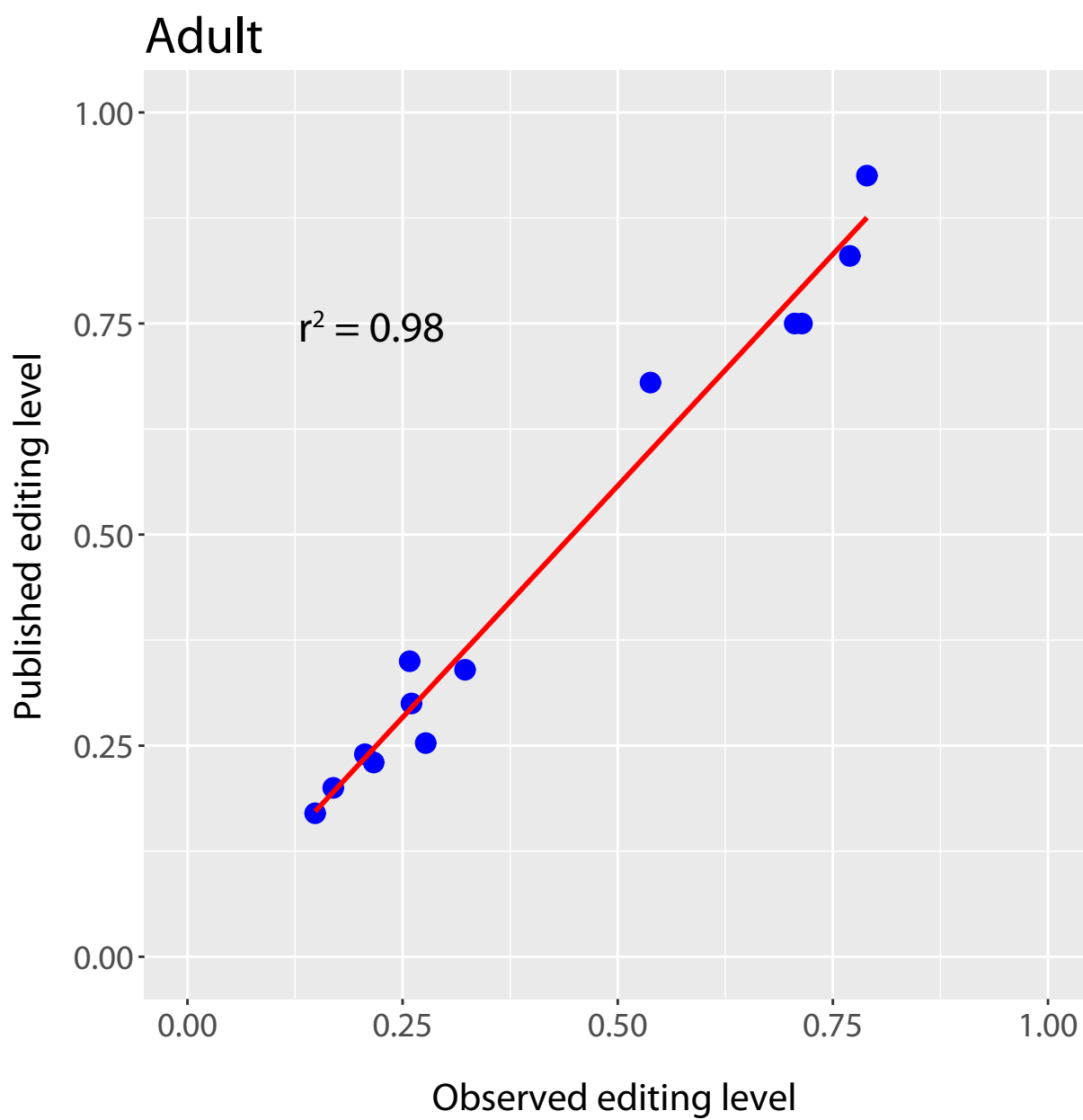

Supplement: Supplementary file 12 — Figure S4. Correlation of observed editing levels to previously published editing levels. Also shown in Additional file 13. [file 12915_2019_736_MOESM12_ESM.pdf]

Figure S5. Regional expression of *Adar1*, *Adar2* and *Adar3*

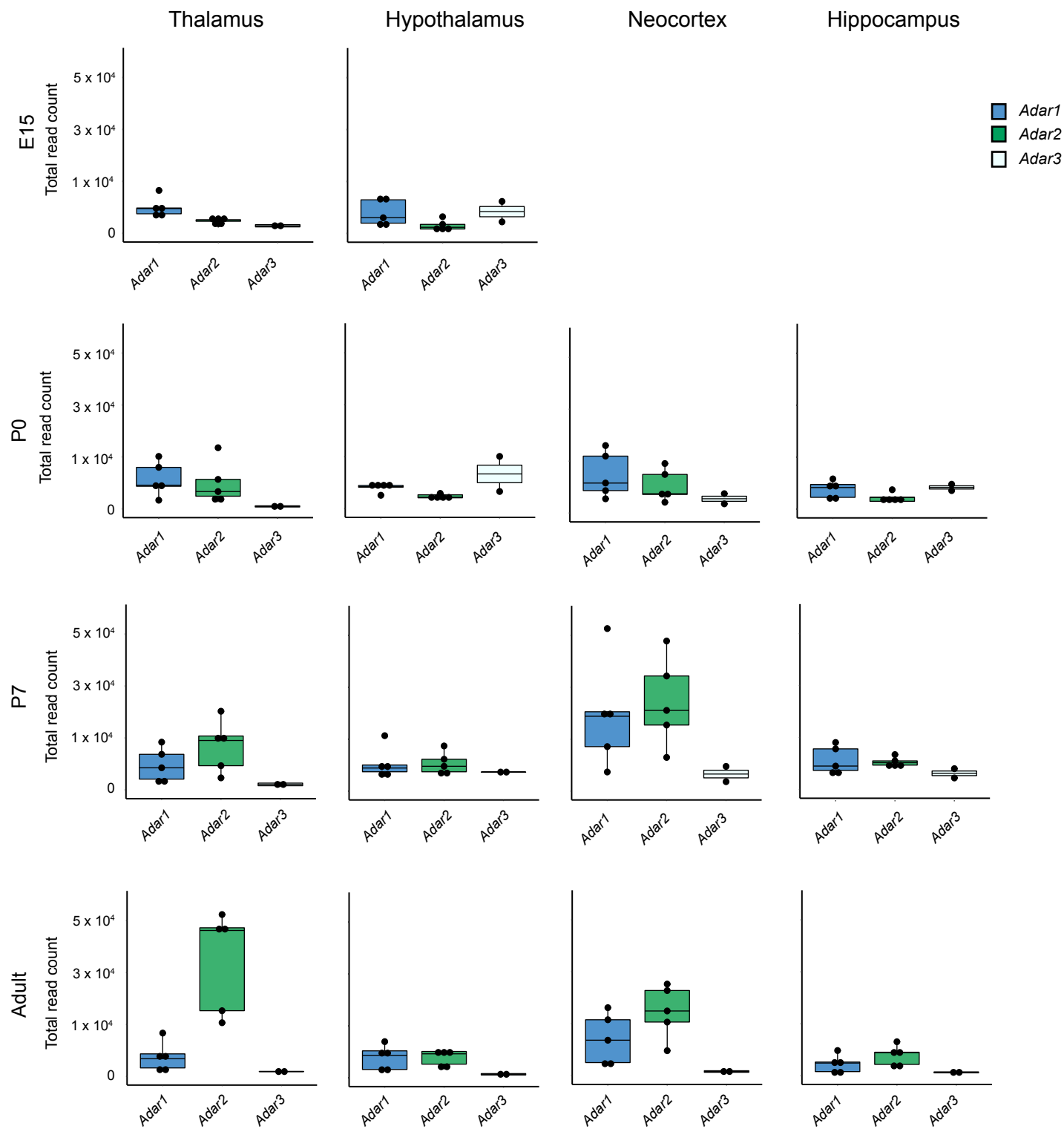

Supplement: Supplementary file 14 — Figure S5. Regional Adar expression. The total number of reads per region for Adar1, Adar2 and Adar3 for each developmental stage (derived from Additional files 7, 8, 9, 10). Each dot represents a replicate. As different developmental stages have different levels of background autofluorescence, resulting in varying quality thresholds for optimal read quality, the read counts for adult are lower than expected. In E15, only the regions thalamus and hypothalamus are presented as neocortex and hippocampus could not be outlined at this stage. [file 12915_2019_736_MOESM14_ESM.pdf]

Figure S6. Adar-specific expression and editing

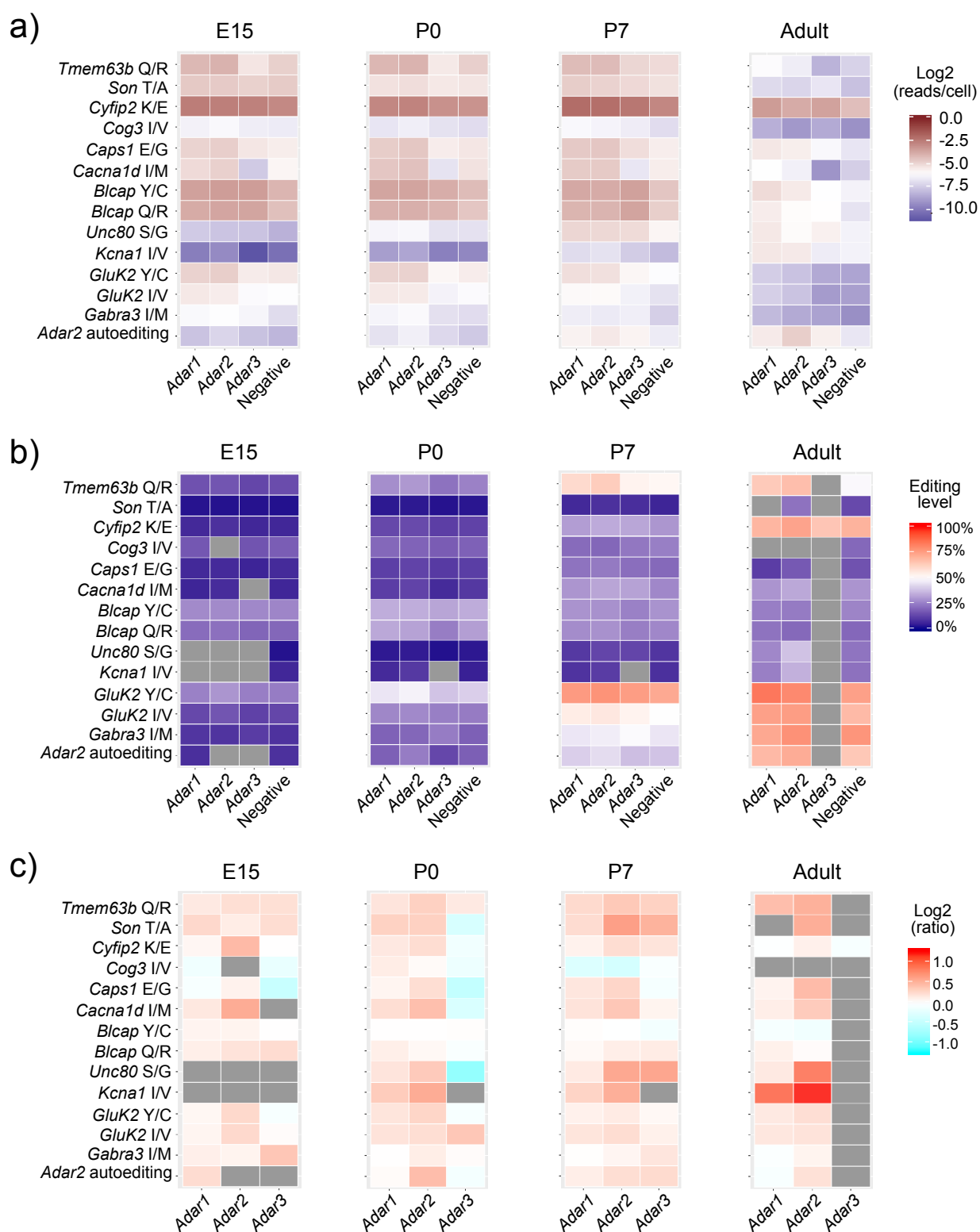

Supplement: Supplementary file 16 — Figure S6. Adar-specific expression and editing. (a) Heat maps showing the Adar-specific expression (reads/cell) for each developmental stage, increasing age from left to right, derived from single cell data (data repository). The color indicates the level of expression from low (blue) to high (red). (b) Adar-specific editing for each developmental stage, presented in heat maps where the color indicates the editing level from low (blue) to high (red). The expression and editing have been calculated based on single cell data for the populations of cells which are positive for Adar1, Adar2 or Adar3 as well as for cells which are negative for all Adar transcripts. (c) Heat maps displaying the editing ratio for each developmental stage. The ratio is calculated as the Adar-specific editing level over the editing level in the Adar negative cells (negative for all Adars). The heatmap color indicates the level of under- (cyan) or over-editing (red). The stronger the color, the larger the deviation from the Adar negative cells. [file 12915_2019_736_MOESM16_ESM.pdf]

Figure S8. Cell segmentation

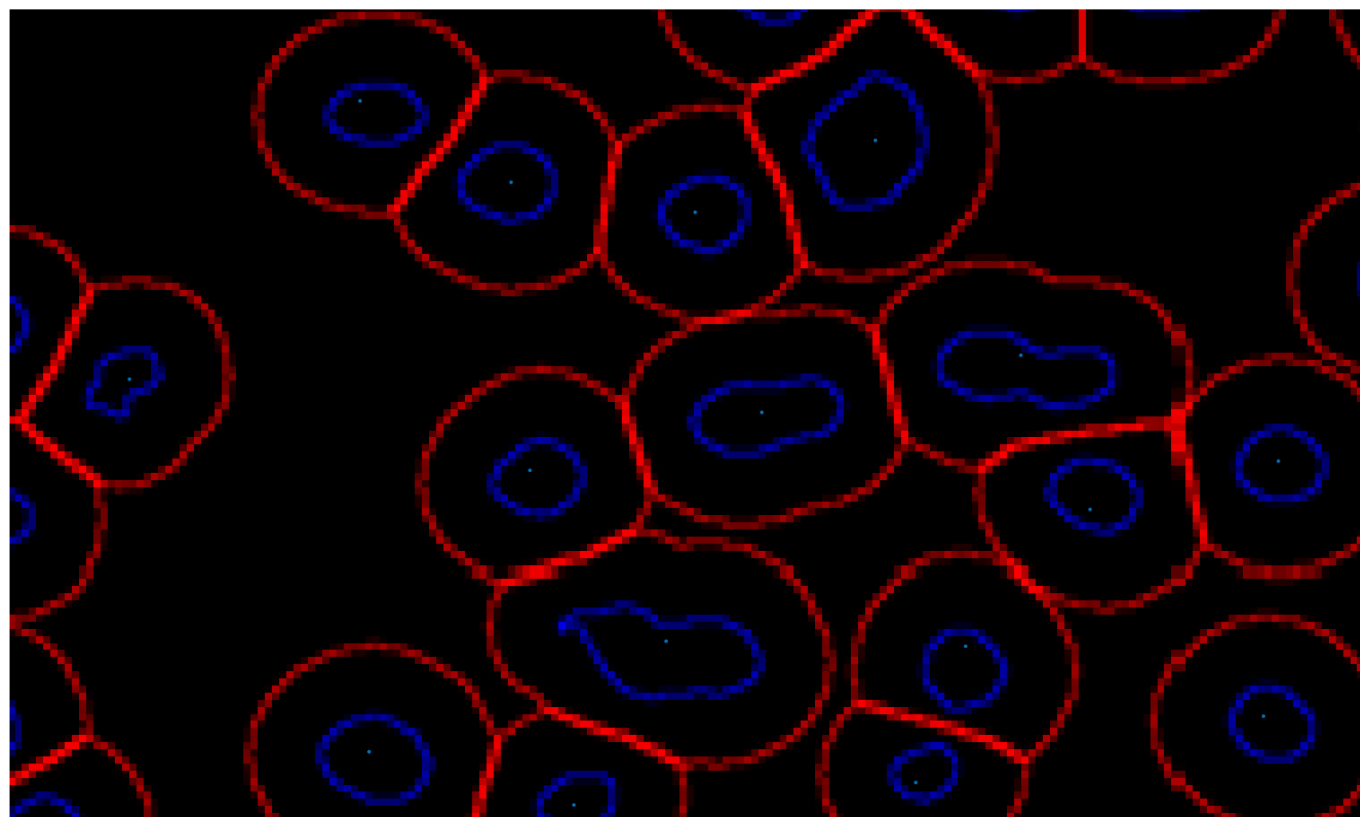

Supplement: Supplementary file 18 — Figure S7. Example of cell segmentation in adult brain. The blue lines mark the outlines of the cell nuclei, based on DAPI staining. The red lines mark the cell border, an approximation of the cell soma based on a fixed distance of 20 pixels from the nucleus border and watershed segmentation to separate cell nuclei in close proximity. [file 12915_2019_736_MOESM18_ESM.pdf]

Figure S7. Marker-specific editing

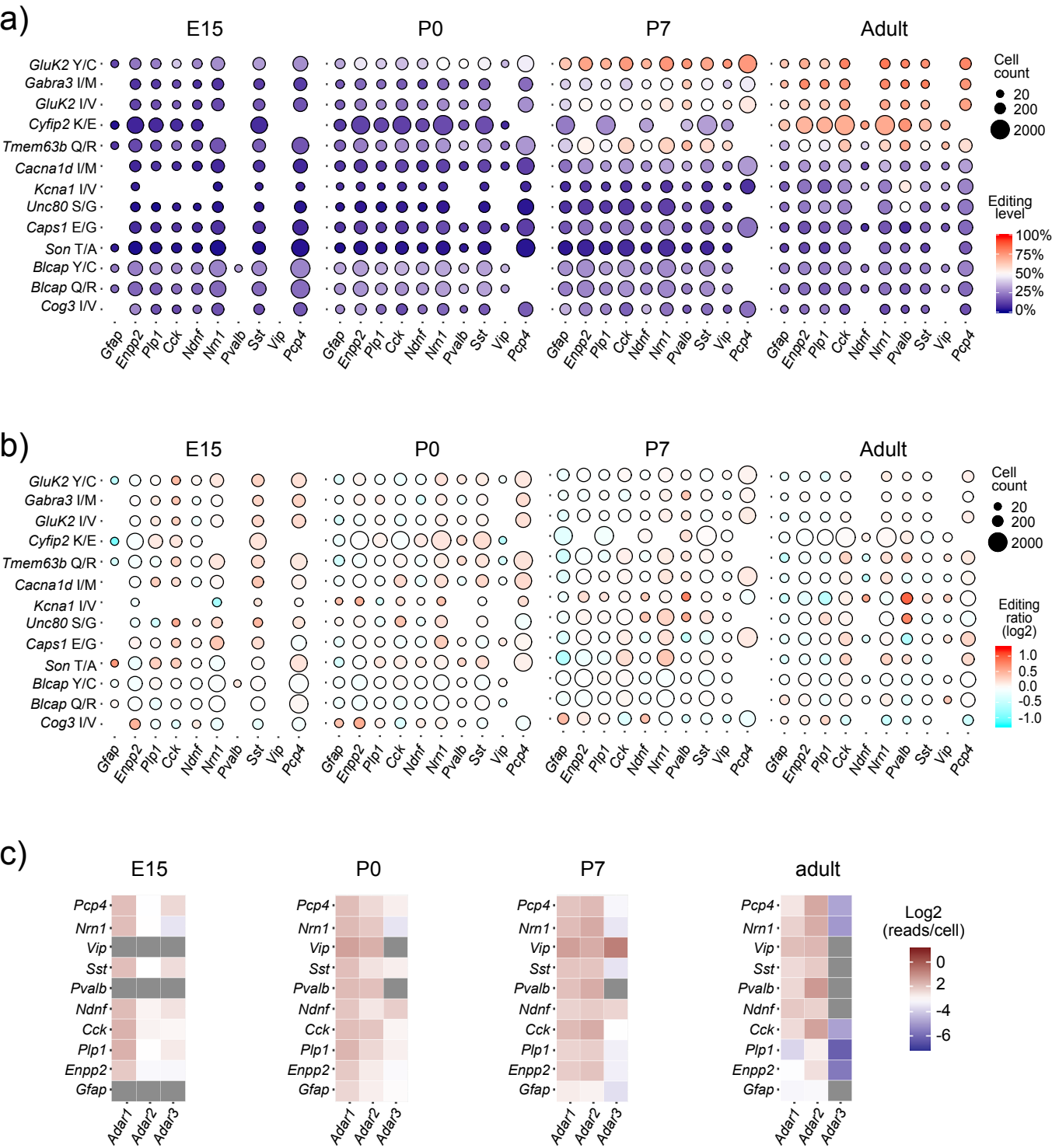

Supplement: Supplementary file 19 — Figure S8. Marker-specific editing. (a) Bubble charts displaying the editing level derived from the population of cells which are positive for a certain marker, based on single cell data (data repository). The color indicates the editing level. (b) Bubble charts of the marker-associated editing ratio, calculated as the editing level associated with a marker over the editing level of the cells negative for that particular marker. The color indicates if the marker-associated editing level is elevated (red), similar to (white) or lower than in other cells. The stronger the color, the larger the deviation. In both (a) and (b), the size of the bubbles indicates the size of the cell population (positive for both the marker and either of the edited or the unedited transcript variant). (c) The marker-specific expression of the ADAR transcripts. The color in the heatmap indicates the expression level of Adar1, Adar2 or Adar3 (log2) in cells positive for the respective markers. Data for marker-specific Adar expression where less than 20 cells were positive for both the marker and the respective Adar are indicated as NA values in grey. [file 12915_2019_736_MOESM19_ESM.pdf]
